# Fan Therapy for Dyspnea in Lung Transplant Recipients in the Intensive Care Unit: A Before-and-After Study

**DOI:** 10.7759/cureus.60029

**Published:** 2024-05-10

**Authors:** Tomoo Sato, Satona Tanaka, Ryuhei Sato, Kentaro Kitao, Shinichi Kai, Chikashi Takeda, Akihiro Ohsumi, Daisuke Nakajima, Koji Egawa, Hiroshi Date

**Affiliations:** 1 Acute Care Nursing Division, Kobe City College of Nursing, Kobe, JPN; 2 Department of Thoracic Surgery, Kyoto University Hospital, Kyoto, JPN; 3 Department of Intensive Care Unit, Kyoto University Hospital, Kyoto, JPN; 4 Department of Anesthesia, Kyoto University Hospital, Kyoto, JPN

**Keywords:** lung transplantation, critical care, intensive care unit, fan therapy, dyspnea

## Abstract

Introduction

Fan therapy has gained attention as a non-pharmacological treatment for alleviating dyspnea in patients receiving palliative care and in those with chronic progressive diseases. However, the effectiveness of fan therapy for dyspnea in critically ill patients in intensive care units (ICUs) remains unclear. This study aimed to investigate the efficacy and safety of fan therapy for lung transplant patients in the ICU.

Methods

Fan therapy was performed on lung transplant recipients (age >18 years) who experienced dyspnea during their ICU stay. A tabletop portable fan was used to blow air on the patient’s face for five minutes providing fan therapy. The intensity of dyspnea before and after the fan therapy was determined, and a statistical analysis was conducted using a paired t-test to evaluate the changes.

Results

Between May 2023 and February 2024, 16 patients who were admitted to the ICU following lung transplantation were screened, and eight patients received fan therapy. Fan therapy was performed at a median of postoperative day 12. Seven patients (87.5%) received mechanical ventilation via tracheostomy. The mean (±standard deviation) numerical rating scale (NRS) for dyspnea before and after fan therapy was 5.6±2.3 and 4.4±1.5, respectively (*p *= 0.08). The mean (±standard deviation) respiratory distress observation scale (RDOS) before and after fan therapy was 4.8 ± 2.0 and 3.8 ± 1.7, respectively (*p *= 0.03). No serious adverse events were observed, and no significant alterations were observed in the respiratory rate, oxygen saturation levels, pulse rate, or blood pressure.

Conclusion

The findings suggest that fan therapy can be safely used to relieve dyspnea in lung transplant recipients during their ICU stay. Further evaluations in larger trials are required to confirm the results of this study.

## Introduction

Dyspnea is defined as "a subjective experience of breathing discomfort that consists of qualitatively distinct sensations that vary in intensity” [1]. It is an important clinical abnormality in various patient populations, including those with cancer, chronic progressive diseases, and lung transplantation [1,2]. Despite medical advances, dyspnea in critically ill patients treated in intensive care units (ICUs) remains a concern. A prospective observational study investigating the prevalence of dyspnea in 96 mechanically ventilated patients in the ICU reported a prevalence of approximately 50%, which could be associated with delayed extubation [3]. Our group’s retrospective observational study of 184 lung transplant recipients in the ICU showed a 63% prevalence of dyspnea, which was associated with a longer time to mobilization and increased ICU stay [4]. Lung transplant surgery involves the dissection of nerves, airways, and blood vessels from the donor's lungs before transplantation. During the lung transplantation, the nerves remain severed while the airways and blood vessels are reconstructed, leading to denervation. This denervation is believed to cause dyspnea by losing the inhibitory effect of the lung stretch receptor input to the brain via the nerves [5,6], which could explain the relatively high incidence of dyspnea in lung transplant recipients. Therefore, the development of interventions to alleviate dyspnea in lung transplant recipients in the ICU is crucial.

Fan therapy is a non-pharmacological intervention that is considered to reduce dyspnea. The mechanism by which fan therapy alleviates dyspnea is unclear; however, it is hypothesized that the stimulation of cutaneous receptors of the trigeminal nerve on the face by blowing air on the face, nose, and mouth may play a role [7-9]. Systematic reviews and meta-analyses have demonstrated the efficacy of fan therapy in alleviating dyspnea in patients with advanced cancer and chronic progressive diseases in palliative and general wards [10-12]. Furthermore, no serious adverse events associated with fan therapy were reported [13,14]. Devices used for fan therapy are affordable and readily available, and their compact design allows easy installation without requiring significant space. In addition, the operation of these fans does not require specialized training, allowing for effortless use by simply turning the fan on and aiming for airflow toward the patient's face. Given the efficacy, lack of side effects, and convenience of fan therapy in reducing dyspnea, its use should be considered in lung transplant patients, where the prevalence of dyspnea is high and the subsequent effects are concerning. However, no studies have evaluated the usefulness of fan therapy in lung transplant patients. We investigated the efficacy and safety of simple fan therapy in a before-and-after intervention study.

## Materials and methods

Trial design

This study was conducted before and after the use of fan therapy for the targeted patients at Kyoto University Hospital’s ICU from May 2023 to February 2024, under trial registration ID UMIN000053103. All the procedures conformed to the principles of the Declaration of Helsinki. The study protocol was approved by the ethics committees (approval number: 22102-12, C1616), and written informed consent was obtained from each patient.

Patients

Regarding the indication for lung transplantation, the criteria proposed by the International Society for Heart and Lung Transplantation were referred to [15], and the indication was discussed by the Ethics Committee of Kyoto University Hospital and the review board in Japan. The inclusion criteria for patients in this study were as follows: (1) experiencing dyspnea at rest, with a subjective score of at least 3 on the numerical rating scale (NRS) (0 = no breathlessness, 10 = worst possible breathlessness); (2) fully conscious and able to communicate (Glasgow Coma Scale (GCS): E4, V5, M6, or Richmond Agitation-Sedation Scale (RASS) = 0); and (3) maintaining a percutaneous arterial oxygen saturation (SpO_2_) of 90% or higher. Exclusion criteria included (1) a temperature exceeding 38.3 degrees Celsius (℃), (2) hemoglobin concentration under 8 g/dl, (3) the presence of delirium (Confusion Assessment Method for the ICU (CAM-ICU) positive), (4) a history of conditions or treatments affecting facial nerves, (5) cognitive deficits, and (6) undergoing rehabilitation within 30 minutes before the intervention. During the study period, all patients aged 18 or older who were admitted to the ICU after lung transplantation were evaluated for participation in this study, and 16 patients were screened.

Interventions

The intervention was uniformly performed on all eligible participants as follows. A tabletop portable fan was started at its lowest speed and was utilized to direct air toward the facial region innervated by the second and third branches of the trigeminal nerve for a duration of five minutes. The distance, position, and speed of the fan were adjusted according to the patient's preferences. The fan model used was HF331NI_WH (Nitori, Toyonaka City, Osaka, Japan), a tabletop portable fan with five blades offering three distinct airflow adjustments, with dimensions of 10 cm × 10 cm × 5 cm. There were no restrictions on the body position or suction during the intervention.

Data collection

Patient characteristics before fan therapy were collected from electronic medical records and included age, sex, body mass index, primary disease leading to transplantation, surgical procedure, donor type, medical history, postoperative days, use of mechanical ventilation, tracheotomy, narcotic administration, and severity score (Sequential Organ Failure Assessment (SOFA)). In addition, data collected before and after fan therapy included the Dyspnea Numerical Rating Scale (Dyspnea-NRS), the Japanese version of the Respiratory Distress Observation Scale (RDOS), anxiety NRS, blood pressure, pulse and respiratory rates, SpO_2_, and facial skin temperature. Information on discomfort due to the fan and eye dryness was also collected after fan therapy. Discomfort due to the fan was assessed using the NRS (Discomfort-NRS). Data before and after intervention were collected by the ICU nurses with 10 years of ICU and 20 years of nursing experience.

Outcomes

The primary outcome was the change in dyspnea before and after fan therapy, which was measured using the Dyspnea NRS for subjective evaluation and RDOS for objective evaluation. The RDOS, developed by Campbell et al. [16], scores eight items (pulse rate, respiratory rate, restlessness, paradoxical breathing pattern, use of accessory muscles for breathing, end-of-breath grunting, look of fear, and expression of fear) on a scale of 0 to 2, with higher total scores indicating more intense dyspnea. RDOS ≥3 signifies that a patient needs relief from dyspnea [17]. Secondary outcomes included respiratory rate, SpO_2_, blood pressure, anxiety NRS score, and facial skin temperature.

Feasibility was assessed based on the number of eligible patients after screening, the number of interventions conducted, and the number of adverse events reported after fan therapy. Adverse events were monitored based on clinical parameters, such as respiratory rate, peripheral oxygen saturation, blood pressure, and pulse rate.

Sample size

Considering the results of the Dyspnea-NRS from previous research [14] and its clinical significance, a sample size of at least six per group was estimated, assuming an effect size of 1.5, an alpha error margin of 0.05, and a statistical power of 80% using a bidirectional hypothesis test. To account for potential dropouts and missing data, at least eight patients per group were recruited.

Statistical methods

Data were analyzed from the collected data by an independent researcher. Statistical analyses were conducted using R software (R Foundation for Statistical Computing, Vienna, Austria). Patient demographics and outcomes were summarized using descriptive statistics, and paired t-tests were used to evaluate dyspnea changes after parametric tests to verify the data distribution. Statistical significance was defined as a *p*-value threshold of less than 0.05.

## Results

Participants

**Figure 1:**
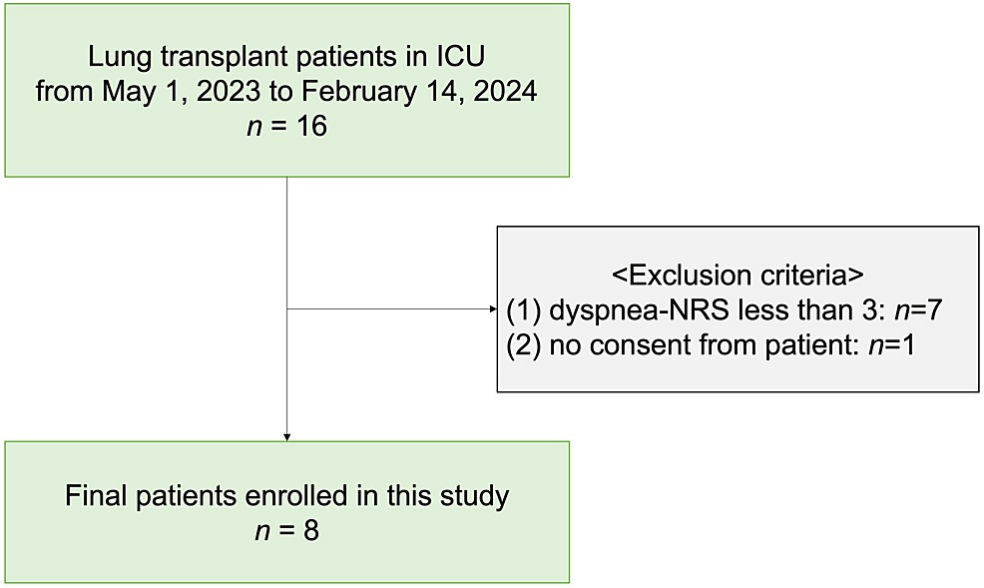
Flow chart of the study Abbreviations: ICU (intensive care unit); NRS (numerical rating scale).

During the period from May 2023 to February 2024, of 16 patients aged 18 or older who were admitted to the ICU after lung transplantation, eight patients were excluded after screening, and fan therapy was performed on eight eligible participants (Figure 1). Details of the patient characteristics are presented in Table 1. The median age of the participants was 35 years. Five participants were female (62.5%). Fan therapy was performed at a median postoperative day 12, with a median SOFA score of 6. Seven participants (87.5%) were under mechanical ventilation through tracheostomy, and one participant (12.5%) was receiving high-flow nasal cannula oxygen therapy. In addition, catecholamines were administered to six participants (75.0%). The ventilator settings and patient positioning remained unchanged, and endotracheal suction was not required throughout the intervention.

**Table 1:** Patients’ characteristics Values are expressed as means with standard deviations in parenthesis, medians with interquartile ranges in parenthesis, or total numbers with percentages in parenthesis. Abbreviations: BMI (body mass index); HSCT (hematopoietic stem cell transplantation); IQR (interquartile range); SOFA (Sequential Organ Failure Assessment).

			Total Sample n = 8
Age, median (IQR)			35 (27.3-43.5)
Female sex, n (%)			5 (63)
BMI, median (IQR). kg/m^2^			17.8 (16.4-19.4)
Pre-transplant diagnosis			
	Idiopathic pulmonary arterial hypertension, n (%)		4 (50)
	Pulmonary complication after HSCT, n (%)		3 (38)
	Interstitial lung disease, n (%)		1 (13)
Type of lung transplant			
	Single, n (%)		2 (25)
	Bilateral, n (%)		6 (75)
Type of donor			
	Deceased-donor, n (%)		8 (100)
	Living-donor, n (%)		0 (0)
Timing of fan therapy, postoperative day, median (IQR)			12 (7.8-13.3)
Condition at fan therapy			
	Tracheotomy, n (%)		7 (86)
	SOFA score, median (IQR)		6 (5-7)
	Respiratory support at fan therapy		
		Ventilator, n (%)	7 (86)
		High flow nasal cannula oxygen, n (%)	1 (13)
	Administration of catecholamines, n (%)		6 (75)

Effectiveness of fan therapy

**Figure 2:**
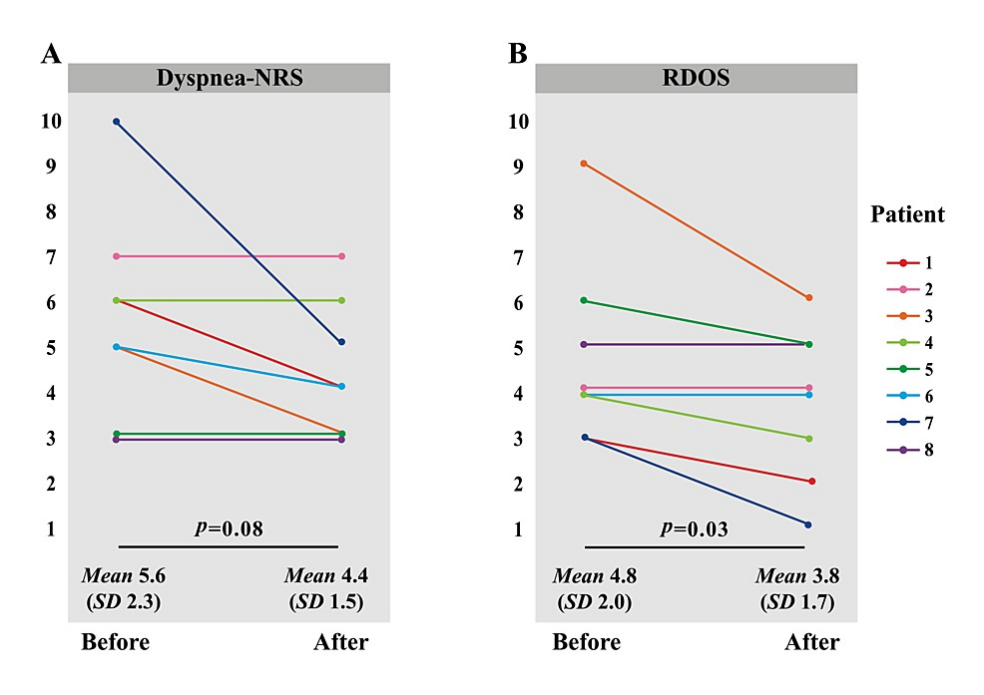
Changes in the dyspnea scores after fan therapy NRS (A) and RDOS (B) scores were recorded before and after fan therapy. The severity of dyspnea before and after fan therapy was evaluated using a paired-sample t-test. Data are shown as the mean ± standard deviation. Abbreviations: NRS (numerical rating scale); RDOS (respiratory distress observation scale); SD (standard deviation).

The impact of fan therapy on dyspnea was evaluated using the Dyspnea-NRS and RDOS, as shown in Figures 2A-2B. The Dyspnea-NRS (mean ± standard deviation) was 5.6 ± 2.3 before fan therapy and 4.4 ± 1.5 after fan therapy (*p *= 0.08). A decreasing trend in the Dyspnea-NRS scores was observed after fan therapy. The RDOS was 4.8 ± 2.0 before fan therapy and 3.8 ± 1.7 after fan therapy (*p *= 0.03). The RDOS was significantly reduced after fan therapy. The observed reduction in each RDOS item was "respiratory rate per minute" in three participants (37.5%), "restlessness" in three participants (37.5%), and "look of fear" in one participant (12.5%) (Table 2).

**Table 2:** Respiratory distress observation scale factors: before and after fan therapy *p*-values for continuous variables were calculated using a paired-sample t-test. Values are expressed as means with standard deviations in parenthesis. Abbreviation: SD (standard deviation).

Variable	Before, Mean (SD)	After, Mean (SD)	*p*-value
Pulse rate	1.0 (0.5)	1.0 (0.5)	-
Respiratory rate	1.5 (0.5)	1.1 (0.6)	0.08
Restlessness	0.6 (0.7)	0.3 (0.5)	0.08
Paradoxical breathing pattern	0 (0)	0 (0)	-
Accessory muscle use: rise in clavicle during inspiration	0.9 (0.4)	0.9 (0.4)	-
Grunting at end-expiration: guttural sounds	0.5 (0.9)	0.3 (0.7)	0.35
Nasal flaring	0 (0)	0 (0)	-
Look of fear	0.3 (0.7)	0 (0)	0.35

The efficacy of fan therapy on anxiety was assessed using the NRS (Table 3). The anxiety NRS was 6.4 ± 1.6 before fan therapy and 6.12 ± 1.7 after fan therapy, showing no significant difference (p = 0.17). A significant decrease in facial surface temperature was observed from 36.8 ± 0.4 ℃ before fan therapy to 36.2 ± 0.3 ℃ after fan therapy (p < 0.01) (Table 3).

**Table 3:** Changes in parameters other than the dyspnea index before and after fan therapy p-values for continuous variables were calculated using a paired-sample t-test. Values are expressed as means with standard deviations in parenthesis. Abbreviations: DBP (diastolic blood pressure); NRS (numerical rating scale); SBP (systolic blood pressure); SD (standard deviation); SpO_2_ (saturation of percutaneous oxygen).

	Before, Mean (SD)	After, Mean (SD)	*p*-value
Anxiety NRS	6.4 (1.6)	6.12 (1.7)	0.17
Facial skin temperature. ℃	36.8 (0.4)	36.2 (0.3)	< 0.01
Respiratory rate. breaths/min	29.8 (6.8)	27.5 (7.5)	0.18
SpO_2_. %	99.4 (1.8)	99.3 (2.1)	0.35
Pulse rate. beats/min	100.6 (8.5)	99.8 (8.1)	0.25
SBP. mmHg	128.3 (15.3)	126.9 (16.3)	0.55
DBP. mmHg	70.5 (17.7)	70.0 (15.3)	0.78

Safety and adverse events

There were no reports of serious adverse events, and no participants showed an increase in Dyspnea-NRS or RDOS scores after fan therapy. No significant changes were observed in the respiratory rate, SpO_2_, pulse, systolic blood pressure, or diastolic blood pressure before and after fan therapy. The discomfort NRS due to fan therapy was 0 in six participants (75%), 2 in one participant (13%), and 4 in one participant (13%). Only one patient (12.5%) complained of dry eyes, but the symptoms promptly subsided following discontinuation of fan therapy without necessitating any medical intervention.

## Discussion

This study evaluated the efficacy of fan therapy for dyspnea in lung transplant recipients during their ICU stay. The study protocol included an objective evaluation of dyspnea, including respiratory patterns and facial expressions, and exhibited marked improvement in these factors after fan therapy. Patient self-reports on dyspnea intensity did not demonstrate a statistically significant reduction; however, Dyspnea-NRS showed a decreasing trend. There were no reports of serious adverse effects following fan therapy or any significant worsening of dyspnea intensity, respiratory rates, SpO_2_ levels, pulse rates, or blood pressure.

In this study, the decrease in the Dyspnea-NRS did not reach statistical significance. By contrast, the RDOS showed a statistically significant decrease. This difference may be attributed to two factors. First, there was a difference in the content of the measurement tools. The Dyspnea-NRS has been validated as a subjective assessment of dyspnea [18], and it is recommended that the NRS be used to assess dyspnea in critically ill patients in the ICU [19]. Nevertheless, given that the Dyspnea-NRS relies on the personal sensations of patients, those undergoing mechanical ventilation could face heightened anxiety, potentially triggering a cycle of dyspnea and fear [4,20,21]. This effect is particularly pronounced in the treatment and environmental stressors within the ICU, contributing to inconsistencies in the responses to personal sensations. The RDOS is an objective dyspnea measurement that includes eight items, such as respiratory rate and accessory muscle use for breathing. Second, the subjective judgments inherent in the evaluation components of the RDOS may have led to evaluator bias. Sakuramoto et al. [22] validated the reliability and validity of the RDOS in Japanese patients. In this study, nurses with 10 years of ICU experience who had received training in the use of the RDOS were asked to score it. A significant decrease in the total RDOS suggested that fan therapy was useful for lung transplant recipients in the ICU to relieve dyspnea. The assessment of dyspnea in critically ill patients is a complex task, and both subjective and objective evaluations are necessary for a more precise and holistic analysis in this type of intervention study.

Fan therapy has been suggested to relieve dyspnea by acting on the insula, anterior cingulate cortex, amygdala, and other brain regions involved in the perception of dyspnea through facial trigeminal nerve stimulation [23]. This study investigated the efficacy of fan therapy in patients receiving mechanical ventilation via tracheostomy and showed that the cooling of facial skin temperature through fan therapy acted on the facial trigeminal nerve, leading to an alleviation of dyspnea. This is consistent with the results of Kako et al., who suggested that decreased facial skin temperature could underlie the dyspnea relief effect of fan therapy [24]. Given the surgical denervation of the pulmonary afferent nerves in lung transplant recipients, neural stimulation of the face using fan therapy may constitute an important aspect of care.

Another important finding of this study was that fan therapy was administered to critically ill patients who received mechanical ventilation and/or catecholamines with no serious adverse events. In addition, the findings demonstrated no adverse effects on dyspnea, respiratory rate, SpO_2_, pulse rate, blood pressure, or other serious adverse events. This is consistent with the findings of previous studies in patients with terminal cancer and advanced disease [10-13]. Discomfort and dry eyes due to fan use have been reported; however, these effects were transient and subsided after fan therapy. Bausewein et al. [25] reported the discomfort caused by fan airflow. To avoid this, fan therapy in this study was initiated at its lowest air volume, with fan distance and speed adjusted according to the patient’s preferences based on previous studies [26-29]. However, ICU patients experience considerable stress due to the treatment and environment [30], suggesting the need for improved methods to minimize discomfort during fan therapy. Dyspnea in critically ill patients may decrease their sense of self-efficacy because they are unable to control it. Fan therapy may increase self-efficacy because it can be initiated by patients themselves, anytime, anywhere [29]. It would be interesting to investigate whether fan therapy can be employed in various critically ill patients treated in the ICU to enhance their recovery.

Limitations

This study has a few limitations. First, the absence of a control group exposed the results to potential regression to the mean and placebo effects, indicating that the findings may be considered preliminary. The choice of a before-and-after study design was based on the anticipated difficulty of recruiting participants for lung transplant studies, where the number of such surgeries is low in Japan. Second, although the number of patients recruited in this study was calculated statistically based on the results of the Dyspnea-NRS from previous research, the small sample size necessitates further randomized controlled trials or studies with larger sample sizes to validate the findings of this study.

## Conclusions

This study suggests that fan therapy is a safe and potentially effective intervention for alleviating respiratory distress in lung transplant recipients in an ICU setting. It is a simple therapeutic approach, and no side effects were found. It can be used with critically ill patients since the results indicated that fan therapy had no adverse effects on dyspnea, respiratory rate, SpO_2_, pulse rate, blood pressure, or other serious adverse events. It can be administered to critically ill patients who received other therapy including mechanical ventilation and catecholamines. In this study, it was shown to be an inexpensive and effective tool, but there were only eight patients and no control group, indicating that further investigation is required. However, due to its potential benefits and lack of side effects, fan therapy can be widely employed to relieve dyspnea in critically ill patients treated in the ICU.
